# 

**Published:** 1842-12-24

**Authors:** 


CONTENTS OF No. 52.

CLINICAL REPORTS: Operation of Lithotripsy.—Breaking of the Instrument in the Bladder—death of the patient. Reported by F. Campbell Stewart, M. D., - - - - - - - - 817

Case of Typhoid Fever followed by Malignant Intermittent—Recovery—Employment of Quinine in large doses. Reported by J. W. Lisle, M.D., Resident Physician, - 819

BIBLIOGRAPHICAL NOTICES: A Treatise on the Diseases of the Eye. By W. Lawrence, F. R. S., etc., etc. From the last London Edition, with numerous additions, and fifty-seven illustrations. By Isaac Hays, M. D., etc., - - - - 822

Elements of Physiology. By J. Müller, M. D., Professor of Anatomy in the University of Berlin, etc. Translated from the German. By Wm. Baly, M. D., Graduate in Medicine in the University of Berlin. Arranged from the Second London Edition. By John Bell, M.D., etc., etc., - 823

The Good Physician; being an Introductory to the Course of Lectures on Materia Medica and Therapeutics, in the Medical Department of Transylvania University, for the Session 1842–3. By Thos. K. Mitchell, M.D., Professor, &c. 824

Introductory Lecture to the Course of Instruction in the Medical Institution of Geneva College. By Thomas Spencer, M. D., Dean, Professor of the Institutes and Practice of Medicine, - - - - - - - - - - 824

Brief Remarks on the Diversities of the Human Species, and on some Kindred Subjects. Being an Introductory Lecture delivered before the Class of the Pennsylvania Medical College of Philadelphia., November 1, 1842. By Samuel George Morton, M.D. - - - - - - - 824

Lectures on the Theory and Practice of Physic. By William Stokes, M.D. And by John Bell, M.D., - - - 830

ANALECTA: Lubaniski on the Urine of Pregnant Women, - - - 832

